# Air Pollution, Kidney Injury, and Green Nephrology—Thinking About Its Association and Causation

**DOI:** 10.3390/jcm14207278

**Published:** 2025-10-15

**Authors:** Sławomir Jerzy Małyszko, Adam Gryko, Jolanta Małyszko, Zuzanna Jakubowska, Dominika Musiałowska, Anna Fabiańska, Łukasz Kuźma

**Affiliations:** 1 Department of Physiology and Pathophysiology, Medical University of Warsaw, 02-091 Warsaw, Poland; smalyszko1999@gmail.com; 2 Department of Invasive Cardiology, Medical University of Bialystok, M. Sklodowskiej-Curie 24a, 15-276 Bialystok, Poland; adamboleslaw@gmail.com (A.G.); kuzma.lukasz@gmail.com (Ł.K.); 3 Department of Nephrology, Dialysis and Internal Medicine, Medical University of Warsaw, 02-091 Warsaw, Poland; zuzanna.jakubowska@wum.edu.pl (Z.J.); anna.fabianska@wum.edu.pl (A.F.); 4 Department of Cardiology, Lipidology and Internal Diseases, Medical University of Bialystok, Żurawia 14, 15-569 Bialystok, Poland; dominika.musialowska@umb.edu.pl

**Keywords:** air pollution, acute kidney injury, acute kidney disease, chronic kidney disease, cardio-kidney-metabolic syndrome, green nephrology

## Abstract

Air pollution is associated with many adverse health outcomes, especially regarding the cardiovascular and pulmonary systems. Recently, the attention of researchers has been attracted to the influence of air pollution on renal function; therefore, more and more data are emerging on the effects of air pollution on kidney diseases. Kidney diseases, especially chronic kidney disease (CKD), are a significant health problem around the world. It is estimated that CKD affects 9.1% of the world’s population, and its prevalence is constantly increasing. CKD is the direct cause of 1.2 million deaths annually. Available experimental models show the relationship between exposure to air pollutants and kidney function. Geographical differences may have an impact on the effect of air pollution on the prevalence of kidney disease. In the majority of studies, long-term exposure to particulate matter-PM_2.5_ is associated with an increased risk of CKD progression to kidney replacement therapy. There is far less evidence on the effect of short-term exposure to air pollution on renal function. Data on the associations between acute kidney injury/acute kidney disease and cardio-kidney metabolic syndrome are even more limited than those on chronic kidney disease. In a mouse model of acute kidney injury, exposure to PM_2.5_ increased susceptibility to chronic kidney disease. In human studies, air pollution was associated with increased risk for first hospital admission for acute kidney injury and mortality due to acute kidney injury. In this review, we would like to summarize the state of knowledge, assessing the influence of air pollution on kidney function. We tried to assess critical associations between air pollution and kidney disease, as well as the translation of these findings in clinical practice. In addition, we aimed to tie green nephrology to air pollution and kidney disease and stressed the paramount role of prevention of kidney disease as the most important aim.

## 1. Introduction

Air pollution is associated with many adverse health outcomes, especially regarding the cardiovascular and pulmonary systems [1,2,3,4,5,6,7,8,9,10,11]. The first scientific statement concerning the influence of air pollution on the incidence of cardiovascular diseases was published in 2004. The expert panel noticed that short-term and long-term exposure to elevated particulate matter influences cardiovascular mortality and morbidity [12]. In 2005, the World Health Organization published the first air quality guidelines, which were updated in 2022 [13,14]. According to the European Environment Agency in 2022, 239,000 premature deaths were recorded in Europe from chronic exposure to fine particulate matter, of which 48,000 were from chronic nitrogen dioxide exposure, and 70,000 were from acute ozone exposure [15].

Polish smog is a specific type of air pollution present in Eastern Poland, which may cause particularly adverse cardiovascular effects. ED-PARTICLE study showed a strong association between air pollution and ischemic stroke, cause-specific mortality in women and the elderly, acute coronary syndromes, atrial fibrillation, and mortality [16,17,18,19,20,21]. Air pollutants affect different organs in many ways, depending on their aerodynamic size. Through the respiratory system, they target different anatomical locations, including the kidneys, among others. To classify air pollutants, particles were divided depending on their aerodynamic size, as thoracic particles (≤10 µm)—PM_10_, fine fraction (≤2.5 µm)—PM_2.5_, coarse fraction (2.5–10 µm)—PM_2.5–10_, and ultrafine particles (<0.1 µm)—UFP [22]. Inhaled particles lead to the release of inflammatory mediators, which result in pulmonary and systemic inflammation [23]. Ambient air pollutants provoke disturbances in the autonomic nervous system and provoke oxidative stress [23,24,25,26]. Airborne particulates, via pulmonary epithelium, may enter the blood circulation and affect tissues directly [27]. Moreover, increased PM_2.5_ concentration relates to hypertension [28,29], and exposure to air pollutants results in a higher risk of diabetes [30,31,32], well-known risk factors of kidney diseases. Chronic kidney disease (CKD), an independent risk factor for cardiovascular disease, is still a huge problem in healthcare worldwide. The median estimated prevalence of CKD in all stages is 9.1% and is still rising [33,34]. It should be emphasized that CKD was the cause of 1.2 million deaths [35]. Moreover, CKD is also recognized as an independent risk factor for the leading cause of deaths globally—cardiovascular diseases [36]—as 1.4 million cardiovascular deaths were attributable to kidney diseases in 2017. It is of great importance to prevent, diagnose early, and treat CKD properly.

In addition to well-known risk factors such as diabetes and hypertension, recently, the attention of researchers has focused on the influence of air pollution on renal function.

Recently, more data have emerged on the effects of air pollution on kidney diseases [37,38,39]. In this review, we would like to present the knowledge about the influence of air pollution on kidney function.

The review was conducted between October 2024 and June 2025. The literature review included publications on research from January 2015 to June 2025. Relevant articles were identified by two authors searching PubMed, Scopus, Web of Science, Embase, and Cochrane Library databases using advanced search and keywords: [[air pollution] OR [air pollutants]] AND [acute kidney injury] OR [acute kidney disease] OR [chronic kidney disease]. The articles were reviewed in three stages by two researchers. We identified 586 entries, 305 of which were rejected because of duplication by automated tools. In the second stage, 180 studies were rejected after reviewing their abstracts due to an ineligible study group or an unsuitable aim of the study. Conference reports and overviews were also excluded. Finally, 101 papers were selected in the third phase. Single case reports did not qualify for the review.

## 2. Air Pollution and Kidney Disease

### 2.1. Experimental Studies

Available experimental data show the relationship between exposure to air pollutants and kidney function. In 2009, Nemmar et al. published the results of the first experimental study investigating in vivo the aggravating effect of pulmonary exposure to diesel exhaust particles (DEPs), in an animal model of acute kidney failure induced by cisplatin [40]. DEPs are the major contributors to PM_2.5_ and ultrafine particles in cities [22]. Six days after the single injection of cisplatin, rats were intratracheally instilled with DEPs or saline (control group). The study group concluded that DEP exposure worsened the renal, pulmonary, and systemic damage in the cisplatin-induced acute renal failure model after 24-h observation [41]. Also, repeated exposure to DEPs in rats with cisplatin-induced kidney damage aggravated cisplatin-induced nephrotoxicity [40].

Also, in 2016, Nemmar et al. [42] published their next experimental study evaluating prolonged exposure to DEPs on mice with chronic renal failure. Kidney failure was induced by adenine added into the feed for 4 weeks, while the DEPs or saline (control) were intratracheally instilled seven times (every 4 days for 4 weeks). They observed aggravated renal oxidative stress and inflammation, as DNA damage occurred after exposure to DEPs in mice with adenine-induced kidney damage [42].

Al Suleimani et al. observed in an animal model that exacerbated vascular damage in rats with adenine-induced chronic kidney failure after exposure to DEPs, if compared to rats that did not undergo intratracheal DEP administration [43].

Waly et al. [44] conducted an in vitro study using human embryonic kidney cells (HEK-293). The study group analyzed the DEP exposure and cisplatin-induced oxidative stress in HEK-293 cells. DEPs augmented the cisplatin-induced HEK-293 cells’ toxic damage. Interestingly, DEPs significantly reduced cysteine uptake. Curcumin, which deserves attention, presented significant protection against DEPs and cisplatin-induced toxicity [44].

**Figure 1:**
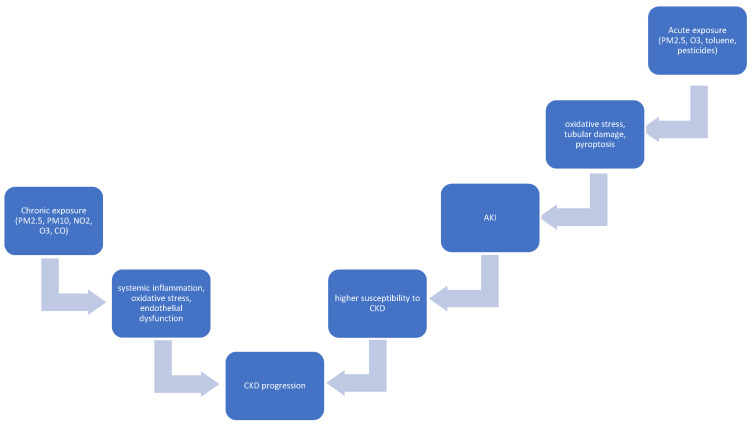
Proposed mechanisms linking air pollution to kidney injury.

Recently, in wild-type C57BL/6J mice exposed to either HEPA-filtered air or clean ultrafine carbonaceous particles (UFP^C^, 450 μg/m^3^) during the prenatal (gestational day 8–9 + 16–17) and/or postnatal (PND 4–7 + 10–13) phase, changes in kidney morphology were observed. The most prominent findings were the alteration of overall areas of the cortex and medulla together, mainly smaller cortical and larger medullary areas. This was associated with alterations in tubular and interstitial structures, mainly with lower tubular area and altered tubular shapes, together with reduced solidity and circularity. These changes may potentially increase kidney vulnerability to damage. It should be stressed that further studies to assess the long-term impact of environmental pollutants on kidney health are of paramount importance [45]. The proposed mechanism of the effects of air pollution on kidney injury is given in Figure 1.

### 2.2. Air Pollution and Kidney Function—Chronic Kidney Disease

Bowe et al. [46] performed a huge prospective observational cohort study consisting of 2,482,737 United States veterans and linked the database with the Environmental Protection Agency database. The study group noticed that a 10-µg/m^3^ increment in PM_2.5_ annual average concentration was associated with increased risk of the occurrence of the following: eGFR lower than 60 mL/min per 1.73 m^2^, chronic kidney disease, eGFR decline ≥ 30%, and end-stage renal disease (ESRD). Subsequently, Bowe et al. [47] also observed that increased risk of CKD, eGFR decline, and ESRD occurrence was also associated with higher annual concentration of PM_10_, nitrogen dioxide (NO_2_), and carbon monoxide (CO).

It was observed, in another study involving 669 US veterans [48], that a 2.1 µg/m^3^ higher 1-year PM_2.5_ concentration was associated with 1.87 mL/min/1.73 m^2^ lower eGFR and an additional annual decrease in eGFR of 0.6 mL/min/1.73 m^2^.

In another study from a US Medicare population of 61,097,767 (all beneficiaries aged 65 years or older), Lee et al. [49] assessed the associations between air pollution and first hospital admission for kidney and total urinary system diseases. They found that positive associations between PM_2.5_ and kidney outcomes persisted at concentrations below national health-based air quality standards.

In a recent study from the USA, Ma et al. [50] investigated the association between long-term exposure to wildland fire smoke PM_2.5_ and nonaccidental mortality and mortality from a wide range of specific causes in all 3108 counties in the contiguous United States, from 2007 to 2020. They reported that long-term wildland fire smoke PM_2.5_ exposure was associated with mortality from various diseases, including chronic kidney disease, cardiovascular diseases, diabetes, etc. In addition, higher mortality was observed in people aged 65 and above. Moreover, Do and Zhang [51] combined the 2023 Centers for Disease Control and Prevention health surveys, criteria air pollutants, and socioeconomic status at the census tract level, and examined the impact of air pollution on human health across the entire U.S. They found that ozone (O_3_) was highly related to the prevalence of cancer and kidney disease, whereas PM_2.5_ was associated with most diseases. They also stressed that minorities and low-income groups across the U.S, exposed to higher levels of PM_2.5_, were more prone to greater health risks, including kidney disease, together with high blood pressure, diabetes, and mental and physical health. Dillion et al. [52], using a random sample of North Carolina’s electronic healthcare records (EHRs) from 2004 to 2016, concluded that one-year average PM_2.5_ was associated with reduced eGFR_cr_, while O_3_ and NO_2_ were negatively associated. On the other hand, Adgate et al. [53] reported that airborne particulate matter exposure in male sugarcane workers appeared to be a risk for chronic kidney disease in Guatemala. Slightly different results were obtained by Yang et al. [54]. In this Taiwanese study, there was no significant association between PM_2.5_ and the prevalence of CKD and eGFR, but increase in average 1-year PM_10_ and PM coarse concentration was associated with a lower eGFR (−0.69 mL/min/1.73 m^2^ for PM_10_ and −1.07 mL/min/1.73 m^2^ for PM coarse) and a higher prevalence of CKD (1.15 for PM_10_ and 1.26 for PM coarse). In another Taiwanese study, 6628 adult patients with CKD were recruited from the Advanced CKD Program in Taiwan between 2003 and 2015. Recently, Chen et al. [55] studied long-term exposure to PM_2.5_ and PM_10_ using high-resolution satellite-based data from the China High Air Pollutants (CHAP) dataset on impact on IgA nephropathy (IgAN). They found that among 1768 biopsy confirmed IgAN patients, 209 of them progressed to ESRD over a median follow-up of 3.63 years. They concluded that higher exposure to both PM_2.5_ and PM_10_ was significantly associated with an increased risk of progression to ESRD, with hazard ratios of 1.62 and 1.36 per 10 µg/m^3^ increase, respectively.

In the most recent study from Taiwan, Wu et al. [56] assessed exposure to air pollution in relation to kidney function decline, measured by ≥30% or ≥40% reductions in eGFR. They designed the study as a nested case–control study using data from the Adult Preventive Healthcare Services database and National Health Insurance claims (2016–2021) with 1-year, 2-year, 3-year, and 5-year follow-up prior to the outcome occurrence. Their cohort consisted of 871,295 health checkup subjects. They assessed air pollution exposure to six pollutants (PM_2.5_, PM_10_, NO_2_, SO_2_, CO, and O_3_) in relation to eGFR decline using land-use regression combined with machine learning algorithms. They found that higher concentrations of all six pollutants were associated with significant increases in the odds of kidney function decline, with CO and PM_2.5_ having the strongest associations with decline in eGFR. The associations were strongest in the 1- and 2-year period relative to 3- and 5-year exposure time. In another study from Taiwan on 9,256,945 participants from Taiwan’s National Health Insurance Research Database (2006 and 2021), Li et al. [57] showed that long-term exposure to ozone was significantly associated with the incidence of CKD, hypertension, and diabetes, as well as transition to multimorbidity and mortality.

In many Chinese studies [58,59,60,61,62,63,64,65,66], there are correlations between air pollution and chronic kidney disease. In a recent cohort study of 47,204 subjects, a 10 μg/m^3^ increase in PM1 (air particles with a diameter ≤ 1 μm) was associated with an increased risk of albuminuria and CKD. These results highlight the critical need for implementing air pollution control strategies to alleviate the burden of CKD [58]. In the most recent comprehensive systemic review and meta-analysis conducted by Li et al [67], 32 studies with 3,022,895 participants were included. They found that long-term exposures to PM_2.5_, PM_10_, and NO_2_ were linked to decreased eGFR. In addition, long-term exposure to PM_2.5_ (per 10 μg/m^3^) was also linked to elevated serum creatinine and uric acid. Moreover, short-term exposure to PM_2.5_ (per 10 μg/m^3^) was also linked to lower eGFR and higher blood urea nitrogen. Based on these data on more than 3 million participants, authors stressed the potential detrimental impacts of ambient air pollution on kidney function.

The most recent study from China [68] investigated the link between indoor air pollution from non-clean fuels used in households and kidney function decline, particularly in a cohort of 4207 middle-aged and elderly individuals in China. Kidney functions were assessed through eGFR (using serum creatinine and cystatin C), and then logistic regression models were used to examine the link between the use of household solid fuel for cooking and the risk of rapid kidney decline and CKD. Tang et al. [68] reported that common use of solid fuel was associated with a higher risk of both rapid kidney decline and CKD. In addition, solid fuels used only for cooking were associated with a higher risk of CKD (OR 1.70; 95% CI: 1.07–2.70). More importantly, switching from solid to clean fuels for cooking was not associated with significant changes in kidney function. The authors also stressed that when solid fuel was used for cooking the risk factors for CKD included lower education, non-smoking status, and being married/cohabiting, whereas when solid fuel was used for heating, risk factors for rapid kidney decline and CKD included being female, having a lower education, being a non-smoker or non-drinker, and being married or cohabiting, as well as having history of gastrointestinal diseases. Hypertension was a risk factor for both rapid kidney decline and CKD with solid fuel use. It is of interest that inhabitants of concrete or steel multi-story buildings using solid fuels had the highest risks of rapid kidney decline and CKD, similar to those in homes smaller than 120 square meters.

Similar data were presented by Thai researchers [69] investigating the spatial–temporal association between PM_2.5_ and its components (organic carbon, black carbon, dust, sulfate, and sea salt) and CKD mortality in Thailand from 2012 to 2021. They found that each 1 µg/m^3^ increase in PM_2.5_, black carbon, dust, sulfate, and organic carbon was significantly associated with increased CKD mortality across 77 provinces. Moreover, they also reported that the geographical difference with the highest death rate was in the Northeast region of Thailand. Air pollution was also a risk factor for renal function deterioration, as defined as sustained eGFR of less than 60 mL/min per 1.73 m^2^ in 1394 South Korean patients with primary glomerulonephritis followed for a mean of 5.1 years [70].

In a recent retrospective study from South Korea, Kwon et al. [71] assessed the effect of air pollutants on the progression of end-stage kidney disease (primary endpoint), as well as on mortality (secondary composite outcome consisting of ESRD and death), in patients with diabetic kidney disease. They pay special attention to medication use. They used the nation-wide forecasted ultra-high-resolution air pollutant data [2.5-μm particulate matter (PM_2.5_), 10-μm particulate matter (PM_10_), nitrogen dioxide (NO_2_), carbon monoxide (CO)] obtained from the Ai-Machine learning Statistics Collaborative Research Ensemble for Air pollution, Temperature, and all types of Environmental exposures (AiMS-CREATE). They updated ambient air pollution data and prescriptions for medication on a monthly basis as time-varying variables in multivariable time-dependent Cox analyses. Their cohort consisted of 9482 patients, followed for a median of 9 years for ESRD and 11 years for composite outcome (ESRD and death). They found that 20.6% of the studied patients progressed to ESRD and 46.7% experienced composite outcomes. They also noted that all four air pollutant concentrations (PM_2.5_, PM_10_, NO_2_, and CO) significantly decreased, with CO showing the most pronounced decline during the follow-up period. In addition, NO_2_ exposure increased ESRD progression risk but was not associated with composite outcomes. Exposure to PM_2.5_ was independently associated with an increased risk of ESRD progression and composite outcomes in patients with DKD, even after comprehensive adjustment of medication used, i.e., renin–angiotensin system blockers. In another study from South Korea, Beak and Yoon [72] analyzed a nationwide sample of 69,066 Korean adults, exploring the association between exposure to air pollutant mixture (PM_10_, PM_2.5_, SO_2_, NO_2_, CO, and O_3_) and renal function expressed as eGFR. They found a negative association between the pollutant mixture and eGFR in the studied population.

Using the Korean National Health Insurance Service and Statistics Korea, Choi et al. [73] in a retrospective cohort study including 2,880,265 individuals (41,501,709 person-years), of which 176,410 were people with disabilities (2,011,231 person-years), analyzed the association between long-term exposure to ambient fine particulate matter (PM_2.5_) and mortality risk in PWD, considering disability type and severity. Patients with kidney impairment showed a significant association between PM_2.5_ and all-cause mortality.

However, geographical differences may have an impact on the effect of air pollution on kidney disease prevalence. In Japan, in Ibaraki prefecture, Nagai et al. [74] studied 77,770 men and women with estimated glomerular filtration rate (eGFR) ≥60 mL/min/1.73 m^2^ who participated in annual community-based health checkups starting in 1993 at 40–75 years old and were followed up through to December 2020. In this first Japanese study, elevated PM_2.5_ was not a significant risk factor for incident CKD.

The first large European study investigating the medium- and short-term impact of air pollutants on kidney function was published in 2021 by Kuzma et al. [75]. A group of 3554 patients was included in the retrospective study. The air pollution data were obtained from the Voivodeship Inspectorate for Environmental Protection. The increase in the annual concentration of PM_2.5_ and NO_2_ was associated with an increased risk of CKD (HR for IQR increase = 1.07 for PM_2.5_ and 1.05 for NO_2_). In the same study, it was observed that increased weekly PM_2.5_ concentration was associated with a reduction in expected eGFR (2% for IQR).

Hamroun et al. [76], in the French REIN registry nationwide cohort study, included 90,373 adult kidney failure patients, initiating maintenance dialysis between 2012 and 2020, and investigated the association of multiple exposures to air pollutants PM_2.5_, PM_10_, and NO_2_ with all-cause and cause-specific death in dialysis patients. They found that long-term multiple air pollutant exposure was associated with all-cause and cause-specific mortality in the dialysis population.

Cesaroni et al. [77] also performed a registry-based study on the association of long-term exposure to nitrogen dioxide (NO_2_), fine particulate matter (PM_2.5_), black carbon (BC), and ozone (O_3_), with end-stage kidney disease incidence in two large population-based European cohorts, i.e., the Austrian Vorarlberg Health Monitoring and Promotion Program (VHM&PP, 136,823 individuals) and the Italian Rome Longitudinal Study (RoLS, 1,939,461 individuals). Interestingly, in the Austrian cohort, no evidence of an association between PM_2.5_ or O_3_ and end-stage kidney disease was observed, whereas in the Italian cohort, PM_2.5_ exposure was associated with the incidence of end-stage kidney disease. Kadelbach P et al. [78], in the European multicenter ELAPSE study on 289,564 persons, followed for 20.4 years, found positive associations between exposure to PM_2.5_- and CKD-related mortality and inverse associations for ozone. This study included only Western European cohorts with at least 10 cases of CKD-associated mortality (Diet, Cancer, and Health cohort from Denmark [DCH], Danish Nurse Cohort [DNC-1993, the prospective sub-cohort of Dutch European Investigation into Cancer and Nutrition [EPIC_NL-Prospect], Etude Epidémiologique auprès de femmes de la Mutuelle Générale de l‘Education Nationale [E3N from France, and Vorarlberg Health Monitoring and Prevention Programme] VHM&PP from Austria). However, after exclusion of the largest sub-cohort contributing 226 cases from Austria, associations became null. There is far less evidence on the effect of short-term exposure to air pollution on renal function.

He et al. [79] observed in multivariable analysis that a higher level of NO_2_ was associated with hospital-acquired acute kidney injury in the Chinese population. The association was present in subgroups depending on age, gender, eGFR, severity of acute kidney injury, and also needed intensive care procedures.

Table 1 and Table 2 show the main outcomes of the published studies on short-term (Table 1 and long-term (Table 2) exposure to air pollutants, presented in ascending order from the least to the most recent.

### 2.3. Air Pollution and Acute Kidney Injury/Acute Kidney Disease

Acute kidney injury (AKI), defined as what was previously called acute renal failure (ARF), is a sudden decrease in kidney function that develops within 7 days, as shown by an increase in serum creatinine or a decrease in urine output, or both [133]. The term acute kidney disease and disorder, abbreviated to acute kidney disease (AKD), has been introduced as an important construct to address this. AKD is defined by abnormalities of kidney function and/or structure with implications for health and with a duration of ≤3 months [134]. Data on associations between acute kidney injury and acute kidney disease are even more limited than those for chronic kidney disease. In an animal model, mice were exposed to urban PM_2.5_ or filtered air for 12 weeks before ischemia–reperfusion injury [135]. Mice showed signs of reduced glomerular filtration, impaired urine concentration ability, and significant tubular damage. PM_2.5_ appears to play a role in sensitizing proximal tubular epithelial cells to ischemia–reperfusion-induced damage, suggesting a plausible association between PM_2.5_ exposure and heightened susceptibility to chronic kidney disease in individuals experiencing acute kidney injury. Similar data were obtained by Hou et al. [136] who assessed the effects of PM_2.5_ exposure in C57BL/6N mice (*n* = 8). They found signs of oxidative stress, autophagy, and pyroptosis. In addition, in vitro studies using HK-2 cells stimulated by PM_2.5_-induced tubulopathy revealed increased reactive oxygen species (ROS) generation, as well as activation of pyroptosis and autophagy.

Bi et al. [81] investigated associations between short-term exposure to PM_2.5_, major PM_2.5_ components [elemental carbon (EC), organic carbon (OC), sulfate, and nitrate], and gaseous co-pollutants (O_3_, CO, SO_2_, NO_2_, and NO_x_) and emergency department visits for kidney diseases during 2002–2008 in Atlanta, Georgia, USA. In addition, Lee et al. [137] studied 61,300,754 beneficiaries enrolled in Medicare Part A fee-for-service (FFS) who were older than 65 years of age and residents of the continental United States from the years 2000 to 2016. In this nationwide population-based longitudinal cohort, exposure to PM_2.5_, NO_2_, and O_3_ was associated with an increased risk of first hospital admission for AKI. More importantly, this association persisted even at low concentrations of air pollution estimated by the National Ambient Air Quality Standard.

He et al. [79] selected a cohort of 11,293 AKI cases from the Epidemiology of AKI in Chinese Hospitalized patients, in which the onset date could be unambiguously determined. In the multivariable analysis, NO_2_ was the sole pollutant associated with the risk of AKI (*p* < 0.001), with a linear relationship to hospital-acquired AKI. Acute renal failure was positively related to 8-day exposure to organic carbon [1.034 (1.005, 1.064)], elemental carbon [1.032 (1.002, 1.063)], nitrate [1.032 (0.996, 1.069)], and PM_2.5_ [1.026 (0.997, 1.057)]. Similarly, Fang et al. [80] reported that PM_2.5_ was inversely associated with eGFR in older Chinese individuals monitored for 72 h.

In South Korea, Min et al. [138] estimated the association between short-term exposure to air pollution (fine particulate matter ≤ 2.5 μm [PM_2.5_] and ozone [O_3_]) and incident AKI by comorbid diseases using the Korea National Health Information Database (NHID). They identified 160,390 incident AKI cases. They found that short-term air pollution exposure to PM_2.5_ was associated with emergency department visits due to AKI. Furthermore, Min et al. [139] investigated the association between short-term exposure to air pollution particulate matter ≤ 2.5 μm (PM_2.5_), ozone (O_3_), and nitrogen dioxide (NO_2_) and AKI-related mortality using a multi-country dataset. They identified 41,379 AKI-related deaths in 136 locations in six countries (Canada, Japan, Portugal, South Korea, Taiwan, and the UK) during 1987–2018.

Their study showed that the risk appeared immediately on the day of exposure to air pollution, gradually decreased, and then increased again, reaching the peak approximately 20 days after exposure to PM_2.5_ and O_3_ [139].

The prospective cohort analysis included 414,885 UK Biobank (UKB) participants who did not exhibit AKI at the study’s outset. Liu et al. [140] assessed the association between prolonged exposure to air pollutants (particulate matter with diameters of 2.5 μm or less (PM_2.5_), between 2.5 and 10 μm (PM_2.5–10_), and 10 μm or less (PM_10_), along with nitrogen dioxide (NO_2_) and nitrogen oxides (NO_x_)) and the risk of AKI and AKI-related death. They also adjusted for potential confounders such as sex, age, ethnicity, education, income, lifestyle factors, and relevant clinical covariates. During follow-up lasting up to 11.7 years, 14,983 cases of AKI and 326 cases of AKI-related death were diagnosed. In addition, they showed that individuals from the higher quartile exposed to higher levels of PM_2.5_, PM_2.5–10_, PM_10_, NO_2_, and NO_x_ had a significantly higher risk of developing AKI and AKI-related death relative to subjects from the lowest quartile (all *p* < 0.05). The relationships between PM_2.5_, PM_2.5–10_, PM_10_, NO_2_, NO_x_, and the risk of AKI showed a significant departure from linearity (*P*_for non-linearity_ < 0.05), whereas the relationships between PM_2.5_, NO_2_, NO_x_, and the risk of AKI-related death did not exhibit a significant departure from linearity (*P*_for non-linearity_ > 0.05) on restricted cubic splines–RCS curves.

In 2022, Lee et al. [83] published an analysis estimating the number of emergency room visits because of the deterioration of kidney function due to short-term exposure to air pollutants (particulate matter ≤ 10 µm, ozone, carbon monoxide, and sulfur dioxide). The highest impact of the increased number of visits to the ER because of AKI in South Korea in the years 2003–2013 was associated with higher ozone concentration. The authors concluded that stricter air quality standards may benefit patients with kidney diseases [83]. In the Table 3 summary of the major studies on air pollution effects on acute kidney injury/acute kidney disease is presented. 

### 2.4. Air Pollution and Cardiovascular-Renal-Metabolic Syndrome

As cardiovascular-renal-metabolic (CKM) syndrome substantially elevates the risk of cardiovascular disease (CVD), Zhao et al. [143] assessed, in a nationwide prospective cohort study, the associations between environmental air pollution and long-term exposure to different sizes of particulate matter (PM_1_, PM_2.5_, and PM_10_) and CVD risk across the four stages of CKM syndrome in a median follow-up of 7 years. They used data from the China Health and Retirement Longitudinal Study (CHARLS, 2011–2018), which included 5824 participants aged 45 years or older with stage 4 CKM according to American Heart Association guidelines. They assessed annual average concentrations of PM_1_, PM_2.5_, and PM_10_ in order to estimate individual exposure and then to assess the burden of air pollution on CVD. They found that increased CVD risk was significantly associated with the highest exposure to PM_2.5_ (HR = 2.31, 95% CI: 2.00–2.66) and rose progressively with CKM stage, being the highest in stage 3 for PM1 (HR = 3.32, 95% CI: 2.24–4.92). Interestingly, patients with CKD had an attenuated association, probably due to the use of nephroprotective drugs. In another study, Shi et al. [144] studied the impact of air pollution on CKM progression in 44,369 (UK Biobank) and 4847 Yinzhou cohort patients (China). The authors found that in the Chinese cohort, there was an association between air pollution and CKM stage progression, whereas in the UK cohort, an association was significant for CKM-related death. Paradoxically, a higher risk for CVD onset and death was reported for non-overweight individuals when exposed to air pollution relative to their overweight counterparts. The authors also underlined the distinct manifestation between these two cohorts and advocated for putting in an effort to reduce air pollution, especially regarding particulate matter in China.

## 3. Pesticides and Acute Kidney Injury and/or Chronic Kidney Disease

Nowadays, evidence is growing on the possible associations between chronic pesticide exposure and adverse health effects, including kidney injury [145,146,147]. Pesticides, along with pollutants, that pollute working environments are xenobiotics, i.e., synthetic substances present in micropollutant concentrations and high concentrations in living organisms as well as in the environment [148]. According to Olowu et al. [149], they are responsible for up to 18% of cases of acute kidney injury in the pediatric population. Glyphosate exposure is linked to the elevated levels of kidney injury molecules in children [150]. Jayasumana et al. [151] demonstrated a link between exposure to xenobiotics, such as organophosphates, paraquat, 2-methyl-4-chlorophenoxyacetic acid (MCPA), glyphosate, bispyribac, carbofuran, mancozeb, and others, and acute renal injury in Sri Lankan farmers.

Detected glyphosate in over >90 of the samples obtained from inhabitants of a rural village in Southern Brazil exposed to pesticide drift from the spraying of neighboring crops [152]. It reflects the widespread contamination of fruits, vegetables, or grains by glyphosate [153]. In two other Brazilian regions with pesticide-intensive use, higher mortality due to AKI was observed, mainly in younger agricultural workers, females, and in the southern region [154,155].

Khacha-Ananda et al. [156] recently reported that farmers who regularly sprayed glyphosate-surfactant herbicides were at high risk of exposure, potentially causing significant kidney injury. Insecticides such as organophosphates and the pyrethroid group are the most used pesticides in agricultural and domestic settings, with malathion being the most commonly used in the US [157] and relatively less toxic than other pesticides [158]. It was reported that malathion exposure caused acute kidney injury with proteinuria/nephrotic syndrome [159,160]. Wan et al. [161] assessed the association between pesticide exposures and the risk of kidney function loss in 41,847 participants using four waves of the National Health and Nutrition Examination Survey (NHANES). They found that exposure to malathion is linked to evidence of altered kidney function. In the study from Almeria, Southeastern Spain, a major hub of greenhouse agriculture, Lozano-Paniagua et al. [162] found that pesticide exposure in the period of its greater use was associated with subclinical tubular damage, which may lead, in time, to chronic kidney disease.

Acute paraquat exposure leading to acute kidney injury has been known since it was first marketed in the 1960s [163,164,165]. However, data on chronic paraquat exposure and kidney disease are limited [151,166,167,168,169]. In endemic areas of unknown chronic kidney disease, urinary glyphosate and paraquat were associated with kidney damage in rural farmers [170]. Similarly, Stem et al. [171] found that certain pesticides were significantly elevated in the urine of sugarcane workers with or without kidney function decline in Guatemala. Finally, increasing paraquat exposure was associated with the incidence of end-stage kidney failure [172]

## 4. Limitations of the Studies

In recent years, there has been growing attention to the effects of air pollution and kidney health. There is evidence that short-term exposure to air pollutants is associated with glomerular filtration rate decline, whereas long-term exposure is related to increased risk of CKD, together with structural damage to kidney tissues. The proposed mechanisms include systemic inflammation, oxidative stress, endothelial dysfunction, and direct nephrotoxicity; however, experimental data are not abundant. In general, data on both short- and long-term effects of air pollution are heterogeneous. There are many cross-sectional and retrospective studies, while prospective studies are very limited, making translation into the real world rather doubtful and challenging. In addition, findings are also not consistent. Most available studies are from Asia and the United States, while data from Europe, Africa, South America, and the Middle East are limited. Future studies in these populations are warranted to assess whether the observed associations are consistent across different ethnic, environmental, and socioeconomic backgrounds. As data from some parts of the world, i.e., South America, Australia, Africa, the Middle East, or even some parts of Europe, are limited or lacking, it is rather a challenge to draw final conclusions regarding the effect of air pollution on kidney health, as well as to propose preventive measures. It appears that heat stress is also an important risk factor for kidney disease, as well, and pollution coming from heating in the winter is even more dangerous. In addition, there is no data available as to precisely when exposure to various air pollutants was assessed in retrospective studies; therefore, it is difficult to estimate the real toxic effect, as concentrations are dependent upon many different factors, including changeable weather conditions, wind, type of heating, traffic conditions, etc.

Therefore, more data are needed to assess more thoroughly and precisely the association between air pollution and kidney health.

## 5. Green Nephrology in Relation to Kidney Disease and Air Pollution

In 2018, an editorial by members of the Executive Council of the European Renal Association—European Dialysis and Transplant Association (ERA-EDTA) was published in Nephrology Dialysis Transplantation as a call for action to medical professionals—both as individuals and as representatives of professional organizations—to contribute to efforts aimed at establishing a ‘greener’ healthcare sector [145]. The key message of the Lancet Countdown was cited in this document [146], particularly Indicator 5.2, which emphasized the importance of research in improving our understanding of the links between climate change and health. On the one hand, it is important to raise awareness among medical professionals of the different components that contribute to the healthcare sector’s carbon footprint. On the other hand, we need to emphasize the links between greenhouse gas emissions, air pollution (especially fine particulate matter, PM_2.5_, sulfur dioxide [SO_2_], and nitrogen dioxide [NO_2_]), and environmental disorders, including kidney disease. Subsequently, an advocacy article by the European Kidney Health Alliance [147] explored the bidirectional relationship between climate change, kidney health, and the environmental impact of kidney care. The authors discussed how the European Green Deal may offer real potential for supporting and galvanizing urgent environmental changes. They also highlighted that exposure to PM_2.5_, SO_2_, and NO_2_—primarily released from fossil fuel combustion—is associated with an increased risk of kidney disease [147]. Their paper underscored the link between air pollution and the rationale for promoting green nephrology. They further emphasized that waste disposal is expensive, with landfills contributing to environmental contamination, incineration both polluting and emitting greenhouse gases, and dialysis therapies themselves consuming large amounts of water and energy [173]. The Italian Society of Nephrology subsequently proposed several approaches to prevent the onset and slow the progression of kidney disease [174]. These include promoting healthier lifestyle choices with added ecological benefits—such as reducing the consumption of red and ultra-processed foods—as well as supporting organic farming and local products, which lower fuel use and pollutant emissions. Very recently, van Vredendaal et al. [175] pointed out the strong consensus among different stakeholders in the kidney health domain on the need for action to better prevent chronic kidney disease, emphasizing the crucial role of the European Union (EU) in providing legislative and financial frameworks to accelerate the transition toward sustainable healthcare in line with the European Green Deal.

## 6. Conclusions

Kidney diseases, especially chronic kidney disease and its complications, pose a global health problem. The literature presented above suggests that air pollution could be considered a risk factor for acute kidney injury and acute kidney disease, both entities promoting the development of chronic kidney disease. Dialysis patients represent a particularly vulnerable group with respect to environmental exposures. In a large nationwide cohort from France, long-term exposure to PM_2.5_, PM_10_, and NO_2_ was associated with increased all-cause and cause-specific mortality in patients receiving maintenance dialysis [76]. The susceptibility of this population may be related to chronic systemic inflammation, oxidative stress, and diminished renal reserve, which amplify the toxic effects of pollutants. Although evidence is still limited, these findings suggest that chronic exposure to common air pollutants is likely to contribute substantially to morbidity and mortality among the dialysis population. Several meta-analyses [37,176,177], large European cohorts, and the UK Biobank [77,122] provide evidence that long-term air pollutant exposure is associated with ESRD risk. In addition, there are studies reporting associations with kidney failure requiring replacement therapy [46,97].

Published results highlight the need for implementing air pollution control strategies to alleviate the burden of CKD, a huge burden on the healthcare system. This is also another argument to intensify our efforts to improve air quality. For the first time, we underlined associations between air pollution, kidney health, and green nephrology as a rationale and way to improve outcomes. However, we also must be aware of the limitations of the published heterogeneous data.

## Figures and Tables

**Table 1:** Studies on the short-term exposure to air pollutants on chronic kidney injury.

	Short/Long-Term	Country/Population	Study Design	Pollutants	Health Effects-Major Findings
1. Fang et al. [80]	Short-term	71 participants/China	longitudinal	PM_2.5_	Significant changes in eGFR were associated with individual PM_2.5_ exposures.
2. Kuźma et al. [75]	Long-term and short-term	3554/Poland	Cross-sectional	PM_2.5_, PM_10_, NO_2_, and SO_2._	The odds of CKD increased with an increase in annual concentration of PM_2.5._
3. Bi et al. [81]	Short-term	306,595 visits/USA	Time-series study	PM_2.5_, major PM_2.5_ components: elemental carbon, organic carbon, sulfate, nitrate, and gaseous co-pollutants (O_3_, CO, SO_2_, NO_2_, and NO_x_)	Positive associations for most air pollutants, for acute renal failure, and positive associations particularly for 8-day exposure to OC, nitrate.
4. Gao et al. [82]	Short-term	2280 older male veterans/USA	Cohort	PM_2.5_, sulfur	Positive relationships of PM_2.5_ mixture with serum uric acid and odds of CKD; sulfur was also associated with a 39% higher odds of CKD.
5. Lee et al. [83].	Short-term	902,043 cases/Korea	Time-series study	PM_10_, SO_2_, CO, O_3_	For all kidney and urinary diseases (902,043 cases), excess ER visits attributable to air pollution existed for all pollutants studied. For AKI (76,330 cases), we estimated the highest impact on excess ER visits from O_3_, while for CKD (210,929 cases), the impacts of CO and SO_2_ were the highest.
6. Wu et al. [84].	Short-term	40,276 CKD-related hospital visits/China	Time-series study	NO_2_	NO_2_ exposure and low temperature were associated with an increased risk of CKD-related hospital visits.
7. Cai et al. [85]	Short-term	101,919 deaths/China	case-crossover s	PM_1_, PM_2.5_, PM_10_, O_3_, NO_2_, SO_2_, CO	All air pollutants were associated with a percent increase in death from kidney disease.
8. Peng et al. [62].	Long-term and short-term	China/2699	Cross-sectional	Ozone (O_3_)	Long-term association between an increment of 3-year ozone exposure with decrease in eGFR, more pronounced in drinkers compared to non-drinkers in relation to ozone exposure.
9. Chu et al. [86]	Short-term	1,209,934 cases/USA	case-crossover study	PM_2.5_, NO_2_ and O_3_	PM_2.5_ exposure associated with acute kidney failure, glomerular diseases, and acute kidney failure; no associations with O_3_ exposure.
10. Chen et al. [87]	Short-term	23,475 GN visits in years 2015–2019	retrospective	CO, NO_2_, PM_10_, PM_2.5_, SO_2_, and O_3_	The risks for GN visits were positively associated with CO exposure.
11. Ma et al. [88]	Short- and long-term	China/6958 PWHAs (people with HIV/AIDS)	Cross-sectional	PM_1_, PM_2.5_, PM_10_	Short-term exposure to particulate matter was related to reduced renal function, mainly PM_1_, PM_2.5_, and PM_10_. Long-term exposure to PM_1_, PM_2.5_, and PM_10_ was positively linked with the incidence of CKD.

**Table 2:** Studies on long-term exposure to air pollutants on chronic kidney disease.

	Country/Population	Study Design	Pollutants	Health Effects-Major Findings
1. Yang et al. [54]	21,656/Taiwan	Cohort	PM_10_, PM coarse, PM_2.5_	An increase in PM_10_ and PM_coarse_ was negatively associated with eGFR and positively associated with the prevalence of CKD; neither outcome was significantly associated with PM_2.5._
2. Bowe et al. [47]	2,010,398/USA	Cohort	PM_10_, NO_2_, and CO	Rise in PM_10_ and CO exposure related to increased risk of eGFR of less than 60 mL/min per 1.73 m^2^, incident chronic kidney disease, and increased risk of end-stage renal disease.
3. Bowe et al. [46]	2,482,737 US veterans	cohort	PM_2.5_	Increase in PM_2.5_ concentration was associated with increased risk of eGFR <60 mL/min per 1.73 m^2^, CKD, eGFR decline ≥ 30%, and ESRD.
4. Kim et al. [89]	24,407/Korea	cohort	PM_10_, NO_2_, SO_2_, CO	Increases in the annual mean concentrations of PM_10_ and NO_2_ were associated with decreases in eGFR levels; no statistically significant association between PM_10_ and NO_2_ concentration and the incidence of CKD.
5. Chen et al. [90]	8,497 adults > 65/Taiwan	Cohort	PM_2.5_, NO_2_	Increments of PM_2.5_ exposure were associated with a lower eGFR.
6. Bragg-Gresham et al. [91]	1,164,057 adults ≥ 65/USA	Cross-sectional study	PM_2.5_	Increase in PM_2.5_ concentration was associated with higher risk of CKD.
7. Chan et al. [92].	100,629/Taiwan	Cohort	PM_2.5_	Rise in PM_2.5_ levels was associated with a higher risk of developing CKD.
8. Blum et al. [93]	10,997/USA	cohort	PM_2.5_	No significant PM_2.5_-eGFR association at baseline. Higher annual average PM_2.5_ exposure was associated with a significantly higher risk of incident CKD.
9. Wang et al. [94]	3622/China	Cross-sectional	PM_10_	Increase in PM_10_ exposure was significantly associated with the increased prevalence of CKD.
10. Ran et al. [95]	61,447/Hong Kong	Cohort	PM_2.5_	Increase in PM_2.5_ concentration related to renal failure and mortality among patients with chronic kidney disease.
11. Lin et al. [96]	161,970/Taiwan	cohort	PM_2.5_, NO, SO_2_	SO_2_, Nox, and NO exposure related to risk of developing CKD and risk of ESRD.
12. Lin et al. [97]	6628 adult with CKD/Taiwan	cohort	PM_2.5_	PM_2.5_ exposure related to progression to KFRT (initiation of maintenance hemodialysis, peritoneal dialysis, or kidney transplantation), no evident association between PM_2.5_ and all-cause mortality.
13. Bo et al. [98].	163,197/Taiwan	Cohort	PM_2.5_	Decrease in the ambient concentration of PM_2.5_ was associated with a 25% reduced risk of CKD development.
14.Feng et al. [99]	820/Belgium	Cross-sectional study	PM_2.5_, black carbon	In a population with moderate exposure, renal function was unrelated to ultrafine particulate.
15. Li et al. [100]	47,204/China	cohort	PM_2.5_	An increase in PM_2.5_ was positively associated with CKD prevalence and albuminuria.
16. Li et al. [101]	80,225/China	Cohort	PM_2.5_, NO_2_, CO, O_3_, SO_2_	An increase in CO and SO_2_ exposure positively associated with CKD. O_3_ exposure was not associated with CKD.
17. Jung et al. [102].	29,602/Korea	Cohort	PM_2.5_, PM_10_, NO_2_, SO_2_, and CO	The significant effects of PM_2.5_ and CO on mortality in CKD patients.
18. Paoin et al. [103]	1839/Thailand	Cohort	PM10, O_3_, NO_2_, SO2, CO	PM_2.5_, NH_4_^+^, NO_3_, OM BC, and SO_4_^2^ exposures associated with risk of CKD.
19. Xu et al. [104]	30,396/Sweden	Cohort	PM_2.5_, PM10, NOx, BC	PM_10_, NOx, and BC exposure were associated with significantly elevated risk for incident CKD; no significant associations with PM_2.5_.
20. Wu et al. [105]	6480/Taiwan	Cohort	PM_2.5_, NO_2_	Increasing PM_2.5_ and NO_2_ level related to risk of eGFR deterioration.
21. Lee et al. [49]	61,097,767/USA	Cohort	PM_2.5_, NO_2_	Annual exposure in PM_2_._5_ and NO_2_ related to risk of total kidney and urinary system disease.
22. Lin et al. [106]	6716/Taiwan	cohort	PM_2.5_, NO_2_, SO_2_	High PM_2.5_ exposure related to significantly increased risk of CKD.
23. Ghazi et al. [107]	20,289 without CKD/USA	Cohort	PM_2.5_	Annual exposure to PM_2.5_ related to developing CKD.
24.Wu et al. [108]	724 in 2020; 758 in 2019/	Cohort	PM_2.5_	In 2020, compared with 2019, reduction in the average PM_2.5_ concentration, and reduction in cumulative days with PM_2.5_ concentration >35 μg/m^3^. From 2019 to 2020, the yearly incidence of eGFR decline ≥5 mL/min/1.73 m^2^ decreased by 1/3. The proportion of patients who started dialysis decreased by 1/3 in 2020 (*p* = 0.001).
25. Oh et al. [109]	15,983/Korea	cross-sectional population-based study	PM_2.5_, PM_10_, NO_2_, and CO	Annual exposure to PM_2.5_, PM_10_, NO_2_, and CO was significantly associated with decreased eGFR. Long-term exposure to PM_2.5_ and PM_10_ was associated with an increased risk of CKD.
26. Huh et al. [110]	134,478 dialysis patients/Korea	Cohort	CO	A significant association between CO exposure and all-cause mortality.
27. Liu et al. [111]	2082/China	Cohort	PM_2.5_ and PM_10_	PM_2.5_ and PM_10_ exposure was associated with an increased risk of CKD.
28. Liu et al. [112]	90,032/China	Cohort	PM_2.5_, PM_10_, NO_2_, SO_2_, O_3_, CO	Combined air pollution associated with risk of CKD.
29. Duan et al. [113]	13,472,425 without CKD/China	Cohort	PM_2.5_	Greater long-term ambient PM_2.5_ pollution is associated with incident CKD.
30. Yang et al. [114]	47,086/China	Cross-sectional study	O_3_	Rise O_3_ concentration was associated with risk of CKD prevalence.
31. Guo et al. [115]	10,942/Taiwan, Hong Kong	Cohort	PM_2.5_, NO_2_ and O_3_	PM_2.5_ exposure was associated with a reduction in the yearly increase in eGFR and a greater risk of incident CKD. Increase in NO_2_ exposure was associated with a higher risk of incident CKD.
32. Luo et al. [116]	1979 patients with IgAN/China	Cohort	PM_2.5_	PM_2.5_ exposure was associated with increased kidney failure risk (ESRD).
33. Chang et al. [117]	5301 CKD patients/Taiwan	Cohort	CO, NO, NO_2_, SO_2_, O_3_, PM_2.5_, and PM_10_	Exposure to CO, NO, NO_2_, SO_2_, PM_2.5_, and PM_10_ associated with a significantly higher risk of renal progression.
34.Wang et al. [118]	458,968/UK	Cohort	PM_2.5,_ PM_10_, NO_2_, and NO	PM_2.5_, PM_10_, NO_2_, and NO exposure are associated with risk for CKD.
35. Hu et al. [119]	5902/China	Cohort	PM_2.5_	PM_2.5_ was associated with the risks of decline of kidney function.
36. Chen et al. [120]	34,088/Taiwan	Cohort	PM_2.5_	Increase exposure of PM_2.5_ associated with higher mortality.
37. Wen et al. [64]	8996/China	Cross-sectional study	PM_2.5_, BC, NH_4_^+^, NO_3_^−^, SO_4_^2−^, OM, O_3_	Long-term exposures to BC and OM were associated with eGFR decline, while O_3_, PM_10_, NH_4_^+^, and NO_3_^−^ were associated with eGFR.
38. Su et al. [121]	26,032 adult/Taiwan	Cohort	PM_2.5_, PM_10_, CO, NO, NO_x_, SO_2_, and O_3_	Elevated levels of PM_2.5_, PM_10_, O_3_, and SO_2_ were associated with a decreased eGFR, whereas higher levels of CO, NO, and NO_x_ were associated with an increased eGFR.
39. Zhang et al. [65]	2,938,653/China	cross-sectional study	PM_2.5_ components: black carbon [BC], organic matter [OM], nitrate [NO_3_^−^], ammonium [NH_4_^+^], sulfate [SO_4_^2−^]	PM_2.5_, NH_4_^+^, NO_3,_ OM BC, and SO_4_^2^ exposures associated with risk of CKD.
40. Li et al. [122]	453,347/UK Biobank	Cohort	PM_2.5_, PM_2.5–10_, PM_10_, NO_2_, and NO_x_	Increase in PM_2.5_, NO_2_, and NO_x_ associated with an elevated risk of incident ESKD. An increased risk of all-cause mortality was associated with PM_2.5_ exposure.
41. Li et al. [63]	367,978/UK Biobank	Cohort	PM_2.5_ and PM_10_, nitrogen dioxide (NO_2_), nitrogen oxides (NO_X_)	Moderate and high exposure to PM_2.5_, NO_2_, and NO_X_ associated with risks of CKD.
42. Zhang et al. [123]	6024 participants/China	Cohort	O_3_	Ozone exposure was negatively associated with the eGFR.
43. Shang et al. [60]	China/1738 patients with T2DM and CKD	Cohort retrospective	O_3_, PM_2.5_, PM_10_, NO_2_, SO_2_ and CO	Association of PM_2.5_ and PM_10_, and CO and SO_2_ concentration with ESRD.
44. Zhang et al. [124]	UK/40,513 diabetic patients	Cohort	PM_2.5_, PM_10_, PM_2.5–10_, NO_2_, and NO_X_	Multiple air pollutants were positively associated with incident CKD in diabetic patients in the UK.
45. Peng S et al. [125]	China/13,041	Cohort	PM_1_, PM_1–2.5_ PM_2.5_, PM_2.5–10_ and PM_10_	Increased risk of kidney disease was associated with PM_1_, PM_1–2.5_ PM_2.5_, PM_2.5–10_, and PM_10_ exposure.
46. Yang et al. [126]	China	cross-sectional	PM_2.5_ components	Significant associations between long-term exposure to three PM_2.5_ components [including black carbon (BC), sulfate (SO_4_^2−^), and organic matter (OM)] and CKD prevalence. No association between [nitrate (NO_3_^−^) or ammonium (NH_4_^+^)] with CKD prevalence.
47. Kim et al. [127]	Korea/61,073	Cohort	O_3_	Long-term ambient O_3_ increases the risk of ESRD and mortality in CKD.
48. Hwang et al. [128]	164,093/Korea	Cohort	PM_10_, SO_2_, NO_2_, CO, and O_3_	Air pollutant exposures including PM_10_, SO_2_, NO_2_, CO, and O_3_ showed no significant association with incident CKD after adjustments for age, sex, household income, area of residence, and the Charlson comorbidity index.
49. Dai et al. [58]	China/81,137	Cohort	PM_2.5_ constituents—black carbon, ammonium, nitrate, sulfate, soil particles, sea salt	PM_2.5_ constituents had positive correlations with CKD as well as black carbon, ammonium, nitrate, organic matter, sulfate, soil particles, and sea salt.
50. Chen R et al. [129]	China/47,204	Survey cross-sectional	PM_1_, PM_2.5_, PM_1–2.5_	Rise in PM_1_ was related to a higher CKD risk and albuminuria (OR, 1.11; 95% CI, 1.05–1.17), but no significant relationship was found for PM_1–2.5_.
51. Dillon et al. [52]	USA/7722	Cross-sectional study	PM_2.5_, NO_2_, O_3_	Positive associations between PM_2.5_, O_3,_ and NO_2_ with CKD; NO_2_ was inversely associated.
52. Zhao et al. [130]	992 T2D patients/Taiwan	Cohort	PM_2.5_, NO_2_	Patients exposed to PM_2.5_ and NO_2_ were found to have an increased risk of CKD occurrence.
53. Kadelbach et al. [78]	Multicentre—Netherlands, Denmark, Austria, France/289,564	cohort	NO_2_, black carbon (BC), O_3_, PM_2.5_	Associations between long-term exposure to air pollution and chronic kidney disease-associated mortality were positive for PM_2.5_, BC, NO_2_, and inverse for O_3_.
54. Nagai et al. [74]	Japan/77,770	cohort	PM_2.5_	Elevated PM_2.5_ did not represent a significant risk factor for incident CKD in Ibaraki prefecture in Japan.
55. Leonetti et al. [70]	Thailand/analysis included 718,686 CKD-related deaths	Spatial–temporal analysis	PM_2.5_ (black carbon, organic carbon, dust, sulfate, and sea salt)	Each 1 µg/m^3^ increase in PM_2.5_, black carbon, dust, sulfate, and organic carbon was significantly associated with increased CKD.
56. Yi et al. [70]	Korea/1394 patients with glomerulonephritis	retrospective cohort	PM_10_, PM_2.5_, CO, and NO_2_	Significant associations between elevated levels of PM_10_, PM_2.5_, CO, and NO_2_ with the progression of kidney disease (GFR < 60), as well as between PM_10_, PM_2.5_, and CO with lower eGFR.
57. Kilbo Edlund et al. [131]	30,154/Sweden	cross-sectional analysis	PM_2.5_, PM_10_, NO	PM_2.5_ exposure was associated with 1.3% (95% CI 0.6, 2.0) higher eGFR per 2.03 µg/m^3^ (interquartile range, IQR). PM_2.5_ exposure was also associated with elevated serum matrix metalloproteinase 2 (MMP-2) concentration; increased filtration is an early sign of renal injury and may be related to the relatively healthy population at comparatively low exposure levels. Furthermore, PM_2.5_ exposure was associated with higher serum MMP-2, an early indicator of renal and cardiovascular pathology.
58.Chin et al. [132]	992 T2D patients/Taiwan	Cohort	PM_2.5_, NO_2_	Patients exposed to PM_2.5_ and NO_2_ were found to have an increased risk of CKD occurrence.

**Table 3:** Studies on air pollution and acute kidney injury/acute kidney disease.

	Short/Long-Term	Country/Population	Study Design	Pollutants	Health Effects
1. Lee et al. [83]	Short-term	902,043 cases/Korea	Time-series study	PM_10_, SO_2_, CO, O_3_	For all kidney and urinary disease, excess ER visits attributable to air pollution existed for all pollutants studied. For AKI, highest impact on excess ER visits from O_3_, while for CKD the impacts of CO and SO_2_ were the highest.
2. He et al. [79]	Short-term	11,293/China	Case-crossover study	PM_2.5_, PM_10,_ CO, NO_2_, SO_2,_ O_3_	NO_2_ is associated with the risk of hospital-associated AKI.
3. Lee et al. [137]	Long-term	61,300,754/USA	cohort	PM_2.5_, NO_2_, and O_3_	Exposure to PM_2.5_, NO_2_, and O_3_ was associated with increased risk for first hospital admission for AKI.
4. Lopez -Bueno et al. [140]	Short-term	Madrid/Spain	retrospective study	PM_10_, PM_2.5_, NO_2_ and O_3_	Extreme heat exacerbates daily emergency hospital admissions due to kidney disease.
5. Min et al. [139]	Short-term	41,379 AKI-related deaths in 136 locations in 6 countries 1987–2018	Case time-series	PM_2.5_, O_3_, NO_2_	AKI-related deaths related to PM_2.5_, warm-season O_3_, and NO_2_.
6. Liu et al. [141]	Long-term	414,885 UK Biobank (UKB) participants	Cohort	PM_2.5_, PM_2.5–10_, PM_10_, NO_2_, NO_x_	Higher risks of AKI for each 5 microgram per cubic meter increase in PM_2.5_ and PM_10_, each 10 microgram per cubic meter increase in NO_2_ and NO_x_, respectively.
7. Min et al. [138]	Short-term	South Korea/160,390 incident AKI cases	Spatial–temporal case-crossover	PM_2.5_, O_3_	Short-term exposure to PM_2.5_ and O_3_ was associated with ED visits due to AKI. Incident Aki was associated with conjunction with ischemic heart disease, cerebrovascular disease, gastrointestinal bleeding, and pneumonia. For O_3_, relevance of AKI with ischemic heart disease.
8. Xiao et al. [142]	Short- and long term	45,249 hospitalized patients/China	case-crossover	CO, PM_2.5_, PM_2.5–10_	CO, PM_2.5_, and PM_2.5–10_ exposure related to significant increase in kidney failure hospitalization, in particular in cold seasons.

## Data Availability

No new data were generated.
